# Development and validation of a risk score to predict neonatal mortality among NICU admissions in Southern Ethiopia: a retrospective follow-up study

**DOI:** 10.3389/fped.2025.1496019

**Published:** 2025-06-12

**Authors:** Shumet Mebrat Adane, Achamyeleh Birhanu Teshale, Daniel Gashaneh Belay, Solomon Gedlu Nigatu

**Affiliations:** ^1^ Department of Epidemiology, College of Medicine and Health Sciences, Hawassa University, Hawassa, Ethiopia; ^2^ School of Public Health and Preventive Medicine, Monash University, Melbourne, VIC, Australia; ^3^ Curtin School of Population Health, Curtin University, Perth, WA, Australia; ^4^ Department of Epidemiology and Biostatistics, Institute of Public Health, College of Medicine and Health Sciences, University of Gondar, Gondar, Ethiopia

**Keywords:** prediction model, risk score, neonatal mortality, neonatal intensive care unit, Ethiopia

## Abstract

**Background:**

The World Health Organization reported 2.6 million neonatal deaths in 2016, accounting for nearly 46% of all under-five deaths globally. Ethiopia is among the top 10 countries with the highest neonatal mortality, with an estimated 122,000 newborn deaths annually. This study aimed to develop and validate a risk score to predict neonatal mortality.

**Methods:**

We conducted a retrospective follow-up study among 845 neonates admitted tot Hawassa University Comprehensive Specialized Hospital, Southern Ethiopia. Data were entered into EpiData version 4.6 and analyzed using R version 4.0.5. Variables with *p* < 0.25 in the bivariable analysis were entered into the multivariable model. A stepwise backward elimination technique with *p* < 0.1 for the likelihood ratio test to fit the reduced model. Finally, variables with *p* < 0.05 were considered statistically significant.

**Results:**

Of the 845 neonates included in the study, 130 died, resulting in a neonatal mortality incidence proportion of 15.4% (95% CI: 13%, 17%). Seven variables, namely, residence, primigravida, low birth weight, amniotic fluid status, Apgar score, perinatal asphyxia, and breastfeeding, were included in the model. The AUC of the final reduced validated model was 0.781 (95% CI: 0.73, 0.82). The accuracy of the model was also assessed by calibration and resulted in a *p*-value of 0.781. The model had a sensitivity and specificity of 80% and 66%, respectively. Decision curve analysis of the model provides a higher net benefit across ranges of threshold probabilities.

**Conclusion:**

We constructed and internally validated a prediction model with good performance. This model is feasible and applicable in healthcare settings to reducing neonatal mortality and improving overall maternal and child healthcare.

## Background

Neonatal mortality (NM) refers to the death of a neonate within the first 4 weeks of life or during the neonatal period and is expressed per 1,000 neonates (1). It is classified into two categories: early neonatal mortality (ENM), which refers to the death of neonates within the first 7 days of life, and late neonatal mortality(LNM), which refers to the death of neonates between 7 and 28 days of life (2).

The World Health Organization (WHO) reported 2.6 million neonatal deaths in 2016, accounting for nearly 46% of all under-five deaths worldwide. Mortality remains the most direct and significant predictor of population-level health; however, 62% of global deaths are unreported, mainly in Africa. With numerous intervention initiatives and policies, the WHO and other stakeholders have worked to minimize maternal and infant mortality (3). Every day, approximately 6,700 neonatal deaths occur, with nearly a third of all neonatal deaths happening on the first day after birth and nearly three-quarters occurring in the first week of life (4, 5).

Globally, NMR was decreased by 51% between 1990 and 2017. However, it remains highest in West and Central Africa and South Asia (6). The annual neonatal mortality rate in the regions (sub-Saharan Africa and South Asia) is more than nine times higher than the average NMR in high-income countries. South Asia and sub-Saharan Africa together accounted for 79% of the overall neonatal death burden, and South Asia alone accounted for 38% of neonatal deaths, and approximately 18% of neonatal deaths occurred in East and South Africa (6). According to the WHO estimates, a significant proportion of all under-five deaths occur in the neonatal or perinatal period (7). Ethiopia is among the six countries which account for half of the global under-five mortality (8–10). It 's one of the top 10 countries with the highest level of worldwide neonatal mortality figures, with an estimated 122,000 newborn deaths per year, which accounts for two-thirds of global neonatal deaths (8–10).

Findings from various studies revealed that prematurity is the major cause of death during the early neonatal period, while asphyxia is the major cause of death during the late neonatal period (11, 12). Additionally, respiratory distress (RD) syndrome is a common cause of neonatal moratlity (11, 13). Other contributing factors associated with both ENM and LNM include birth injury, hyperthermia, neonatal infections, and birth asphyxia (14–16).

Other factors associated with NM include maternal age, parity, pregnancy interval, multiple pregnancies, maternal health problems during pregnancy, malpresentation, problems during delivery, and infant sex (17, 18).

Efforts have been made globally and nationally to reduce this devastating public health problem, which includes Millennium Development Goals (MDGs) later extended to Sustainable Development Goals (SDGs) (19). Ending preventable child deaths is a critical part of the Global Strategy for Women’s, Children’s, and Adolescent’s Health (2016–2030) and the third Sustainable Development Goal (SDG) to ensure safe lives and encourage prosperity for all citizens of all ages (20).

Over the past two decades, the Government of Ethiopia has implemented a health sector development program aimed at improving the accessibility and quality of health services for all segments of the population. As a result, there have been significant increases in institutional deliveries, antenatal care (ANC) coverage, and postnatal care provided within 48 h of delivery (21). Ethiopia has adopted and implemented various strategies to reduce maternal and neonatal mortality, such as emergency obstetric and newborn care, adapted to enhance neonatal and maternal outcomes, resulting in substantial success in reducing under-five mortality (22, 23). However, reduction in neonatal mortality has been the least significant (24).

A clinical prediction model combines various risk factors to estimate the probability of mortality in newborns In neonatal care units. This facilitates personalized diagnostic and therapeutic decision-making within healthcare settings and enables the early identification of high-risk neonates, ensuring timely and appropriate interventions (25–27).

A risk score model derived from clinical variables can support evidence generation and facilitate timely decision-making. Moreover, up to our knowledge in the study area, a study conducted regarding NM particularly did n't consider a prediction model. Validation of such model analysis plays a great role in identifying problems and allowing accurate risk estimation. Therefore, this study aimed to identify important predictors that can forecast which infants are at high risk of neonatal death and which are not. This helps policymakers ad governmental and non-governmental organizations to take appropriate interventions.

## Methods

### Study design and period

An institution-based retrospective follow-up study was conducted among neonates admitted to the neonatal intensive care unit (NICU) from 1 February 2016 to the end of January 2021 at Hawassa University Comprehensive Specialized Hospital (HUCSH). Data were extracted from 1 April to 10 May 2021.

### Study area

The study was conducted at HUCSH, the largest comprehensive specialized hospital in Southern Ethiopia, with over approximately 400 beds, serving residents of the region as well as people from the surrounding region. It 's approximately 275 km south of Addis Ababa and has a population of 157,879 according to the 2007 census. HUCSH, Adare General Hospital, and Tulla Primary Hospital are public hospitals located in Hawassa City. Currently, HUCSH serves over 20 million people. Before 2014, NICU services were provided in combination with the pediatric ward. After that, it is provided separately. It has 33 beds, five kangaroo mother care (KMC) beds, and four mother-side beds. It also has 13 radiant warmers and four incubators. Thermoregulation, blood transfusions, tube feedings, phototherapy, intravenous fluids, antibiotics, and continuous positive airway pressure are among the services delivered to neonates. Four pediatricians, 29 residents, 30 BSc nurses, and intern students provide service in the unit.

### Source population and study population

The source population comprised all neonates admitted to the NICU of HUCSH from 1 February 2016 to the end of January 2021.

### Inclusion and exclusion criteria

#### Inclusion criteria

All neonates admitted to the NICU of HUCSH from 1 February 2016 to the end of January 2021 were included in this study.

#### Exclusion criteria

Neonates whose charts had unknown outcome status were excluded.

### Sample size calculation

The sample size estimation was performed using the prediction model sample size calculation technique. There is a limit as to how many candidate predictor variables can be included in the modeling phase. A model that consists of too many predictors is more likely to be overfitted.

The sample size calculation is performed based on an aforementioned “rule of thumb,” which is at least 10 events per predictor. A simulation study examined and concluded that no major problems occurred for 10 events per variable or more is a useful guide to developing/validating a model on a given cohort (28, 29).

Therefore, we considered 18 predictor parameters in the modeling process. From the previous study, the incidence proportion of neonatal mortality was 21.3% (13).

According to the rule of thumb:

*N* = (*n* × 10)/*I*, 18 × 10 = 180 events need to be observed in the data.

*N* = event needed/proportion of the event; *N* = 180/0.213 = 845.

where *N* is the required sample size, *n* is the number of variables to be tested, and *I* is the incidence of neonatal mortality.

Therefore, we extracted 845 charts of neonates, who were admitted to the NICU of HUCSH from 1 February 2016 to the end of January 2021.

### Sampling technique

The neonatal chart numbers were obtained from the NICU register and Health Management Information Systems (HMIS) database, and prepared frames in Excel were collected from the liaison office. After we found the neonatal registry numbers, the 845 study participants were selected based on simple random sampling techniques by using SPSS version 24 software.

### Study variables

#### Dependent variable

The dependent variable was neonatal mortality (yes/no) in the NICU.

Neonatal mortality is defined as the death of neonates within 28 days after birth.

#### Independent variables

Sociodemographic factors include maternal age, residence, age of neonate, and sex of the neonate.

#### Antepartum and maternal-related factors

Pregnancy status, gravidity, parity, ANC visit, illness during pregnancy, history of adverse outcome, duration of pregnancy, syphilis status, and HIV status.

##### Neonatal factors

Apgar score, breastfeeding, birth weight (BW), newborn resuscitation, initiation of breastfeeding, and admission diagnoses such as respiratory distress, perinatal asphyxia (PNA) (the failure of the neonate to initiate and sustain breathing) (30), sepsis, congenital malformation, hyaline membrane disease (RDS), jaundice, hypothermia, prematurity, and meconium aspirated syndrome.

Initial vital signs upon NICU admission were categorized according to WHO definitions.

##### Intrapartum factors

Mode of delivery, place of delivery, amniotic fluid status (AF), presence of meconium, and complications during delivery.

### Data collection and quality control

A data extraction checklist was developed based on relevant literature and the study objectives. It was prepared in English, and data were extracted by trained individuals. A 1-day training was given to data collectors and supervisors, covering the objective of the study, data collection, and supervision of the data collection process.

Data were collected by three health professionals (three diploma nurses with experience in the area), and one BSc nurse supervised the data collection process with the principal investigator using the tools. A filled checklist was checked daily for completeness, accuracy, and consistency. Every morning, the data collectors, supervisor, and principal investigator discuss the previous day's data collection process.

Moreover, we imputed prediction variables missing values by using “MICE” R packages. Imputed variables included birth weight, amniotic fluid status, gravida, Apgar score, and breastfeeding status [10 (1.2%), 3 (0.4%), 2 (0.2%), 48 (5.7%), and 2 (0.2%), respectively]. In contrast, variables missing 15% or more of data were excluded from the analysis. Breastfeeding initiation in an hour was also excluded since it has a missing value of 276 (33%).

### Data processing and analysis

Data were cleaned, coded, and entered into EpiData version 4.6 software and exported to R statistical programming language version 4.0.5 software for further processing and analysis.

Descriptive findings were presented in tables, figures, and text form. The incidence of neonatal mortality was also calculated. Bivariable logistic regression analyses were performed to identify potential determinants associated with neonatal mortality and to select candidate determinants for the multivariable analysis.

We selected the variables with *p* < 0.25 in the bivariable analysis to the multivariable model. A stepwise backward elimination technique with *p* < 0.1 was applied for the likelihood ratio (LR) test to fit the reduced model. The model is validated with the Hosmer–Lemeshow test. Statistical significance was declared at *p* < 0.05.

The validity of the prognostic models was assessed in terms of calibration and discrimination. Calibration was assessed graphically with a calibration plot (31, 32). Discrimination refers to the ability of the model to distinguish between neonates who died and those who survived. This was assessed using the area under the receiver operating characteristic curve (ROC) (33) for model accuracy and discriminative power of predictor variables of neonatal mortality.

We computed ROC and generated calibration plots using the “classifierplots” and “givitiR” packages of R, respectively (34). Coefficients were divided by the lowest coefficients, the finding was transformed into a round number (nearest integer), and then the prediction model was developed by using the prediction score. We determined the total score for everyone by assigning the points to each variable present and adding them up. Moreover, sensitivity, specificity, positive predictive value (PPV), and negative predictive value were calculated using the optimal cutoffs of levels which were executed by the Youden index method.

Using the bootstrapping technique (35), AUCs were internally validated. A total of 10,000 random bootstrap samples with replacements were drawn from the data set. The model's predictive performance after bootstrapping is deliberated as the performance that can be expected when the model is applied to other similar populations.

The clinical and public health impact of the model was evaluated using decision curve analysis (DCA) (36), of standardized net benefit across a range of threshold probabilities (0–1). In the DCA, the model was compared against two extremes: “intervention for all” and “no intervention.”

## Results

### Sociodemographic characteristics

A total of 845 charts of neonates admitted to the NICU of HUCSH were reviewed. The median age of the mothers was 26 (IQR: 24, 30) years, and nearly two-thirds [550 (65%)] of mothers were in the age group of 20–29 years. Nearly half [435 (51.5%)] of mothers were from rural areas (Table 1).

**Table 1:** Sociodemographic characteristics of neonates admitted in the neonatal intensive care unit of Hawassa University Comprehensive Specialized Hospital, southern Ethiopia, 2021**.**

Variables	Category	Death		Total *n* (%)
		Yes, *N* (%)	No, *N* (%)	
Maternal age	<20	6 (4.6%)	30 (4.2)	36 (4.4)
	20–29	78 (60%)	472 (66.0)	550 (65)
	≥30	46 (35.4%)	213 (29.8)	259 (30.6)
Residence	Urban	39 (30)	371 (51.9)	410 (48.5)
	Rural	91 (70)	344 (48.1)	435 (51.5)
Sex of neonate	Male	75 (57.7)	405 (56.6)	480 (56.8)
	Female	55 (42.3)	310 (43.4)	365 (43.2)
Age of neonate	<7 days	115 (88.5)	610 (85.3)	725 (85.5)
	≥7 days	15 (11.5)	105 (14.7)	120 (14.2)

### Obstetrical factors among mothers of neonates admitted to the NICU

Approximately 807 (95.5%) of mothers attended ANC in the health institution, and 46 (5.4%) had experienced illness during pregnancy. More than two-thirds [583 (69%)] of mothers were multigravida. Approximately 295 (34.9) neonates were delivered by cesarean section. Among all admitted neonates, 824 (97.5%) were born from health institutions. More than one-tenth [115 (13.6%)] of neonates had complications during labor and delivery. A substantial number of neonates [753 (89.1%)] were delivered through cephalic presentation (Table 2).

**Table 2:** Obstetrical factors among mothers of neonates admitted in the Neonatal intensive care unit of Hawassa University Comprehensive Specialized Hospital, southern Ethiopia, 2021***.***

Variables	Category	Death		Total *N* (%)
		Yes, *N* (%)	No, *N* (%)	
Gravidity	Primigravida	32 (24.6)	230 (32.2)	262 (31)
	Multigravida	98 (75.4)	485 (67.8)	583 (69)
ANC follow-up	Yes	118 (90.7)	689 (96.4)	807 (95.5)
	No	12 (9.3)	26 (3.6)	38 (4.5)
Illness during pregnancy	Yes	6 (4.6)	40 (5.6)	46 (5.4)
	No	124 (95.4)	675 (94.4)	799 (94.6)
Mode of delivery	Vaginal	93 (71.5)	457 (63.9)	550 (65.1)
	CS	37 (28.5)	258 (36.1)	295 (34.9)
Place of delivery	Home	7 (5.4)	14 (2)	21 (2.5)
	Health institution	123 (94.6)	701 (98)	824 (97.5)
Presentation	Cephalic	113 (86.9)	640 (89.5)	753 (89.1)
	Non-cephalic	17 (13.1)	75 (10.5)	92 (10.1)
PROM	Yes	12 (9.2)	70 (9.8)	82 (9.7)
	No	118 (90.8)	645 (90.2)	763 (90.3)
AF status	Clear	112 (86.2)	646 (90.3)	758 (89.7)
	Stained	18 (13.8)	69 (9.7)	87 (10.3)
Complication during labor	Yes	18 (13.8)	97 (13.6)	115 (13.6)
	No	112 (86.2)	618 (86.4)	730 (86.4)

AF, amniotic fluid; ANC, antenatal care; CS, cesarean section; PROM, premature rupture of membranes.

### Fetal and neonatal characteristics of the neonates admitted to the NICU

Among the neonates admitted to the NICU, more than half [480 (56.8%)] were males. The median age of the neonate was 1 (IQR: 2) day. More than three-fourths [725 (85.5%)] of neonates aged <7 days. More than two-thirds [592 (70.1%)] of neonates were delivered at term. Among all admitted neonates, nearly two-thirds [531 (62.8%)] of neonates had normal birth weight. A higher number of admitted neonates [738 (87.3%)] had a poor (<7) 5th-minute Apgar score. Among the neonates admitted to the NICU, 775 (91.7%) were breastfeeding at admission. In addition, 103 (12.2%) neonates had respiratory distress (Table 3).

**Table 3:** Fetal and neonatal characteristics of the neonates admitted to the neonatal intensive care unit of Hawassa University Comprehensive Specialized Hospital, southern Ethiopia, 2021.

Variables	Category	Death		Total *N* (%)
		Yes, *N* (%)	No, *N* (%)	
Gestational age	<37 weeks	64 (49.2)	189 (26.4)	253 (29.9)
	≥37 weeks	66 (50.8)	526 (73.6)	592 (70.1)
Birth weight	NBW	53 (40.8)	478 (66.8)	531 (62.8)
	LBW	77 (59.2)	237 (33.2)	314 (37.2)
Apgar score (5th)	<7	87 (66.9)	651 (91.1)	738 (87.3)
	≥7	43 (33.8)	64 (8.9)	107 (12.7)
RD	Yes	26 (20)	77 (10.8)	103 (12.2)
	No	104 (80)	638 (89.2)	742 (87.8)
PNA	Yes	35 (26.9)	80 (11.2)	115 (13.6)
	No	95 (73)	635 (88.8)	730 (86.4)
Sepsis	Yes	28 (21.5)	236 (33)	264 (31.2)
	No	102 (78.5)	479 (67)	581 (68.8)
Breastfeeding	Yes	101 (77.7)	674 (94.3)	775 (91.7)
	No	29 (22.3)	41 (5.7)	70 (8.3)

Apgar, appearance, pulse rate, grimace, activity, respiration; LBW, low birth weight; NBW, normal birth weight; PNA, perinatal asphyxia; RD, respiratory distress.

### Incidence of neonatal mortality

**Figure 1:**
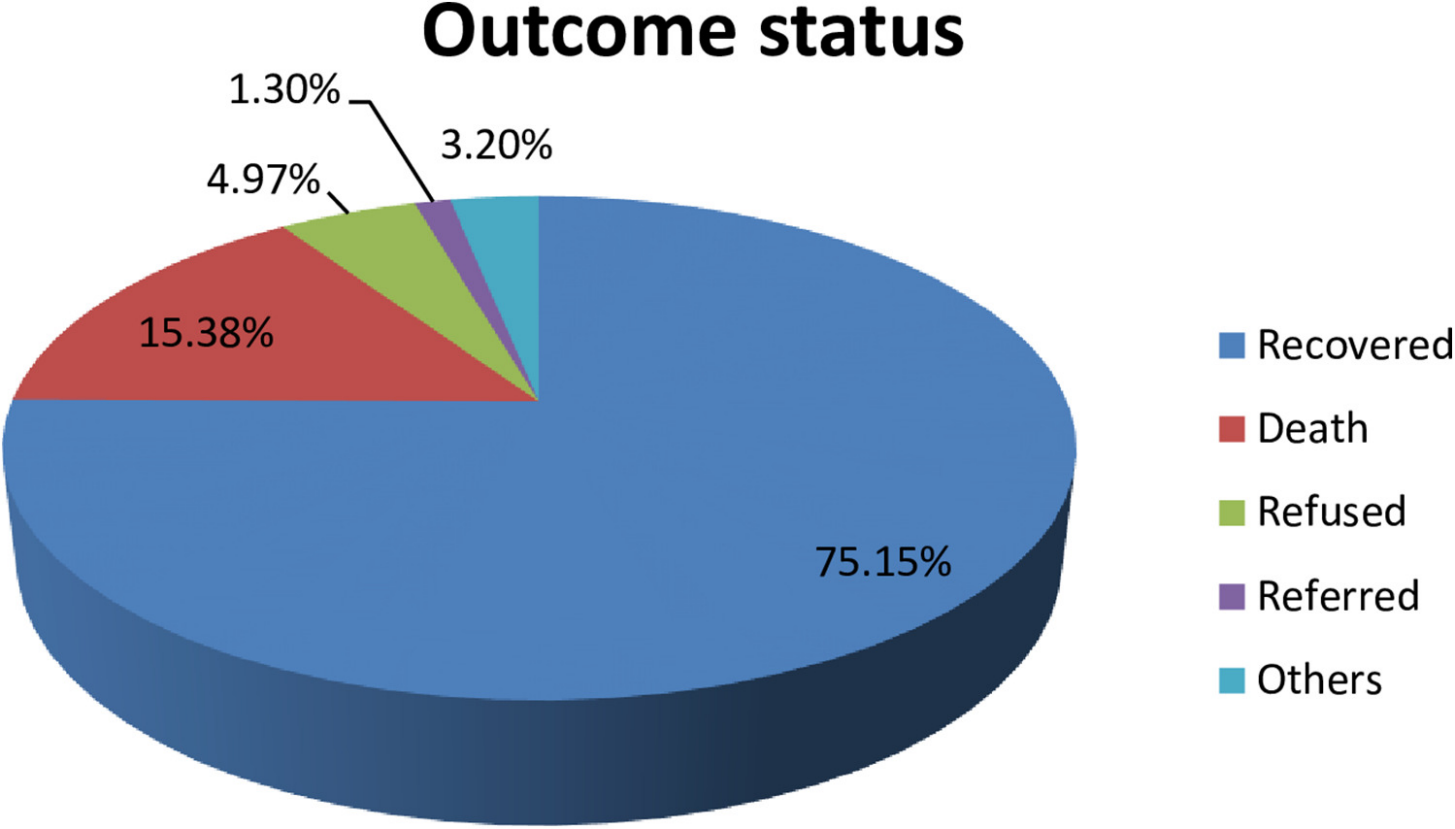
The percentages of outcome status of neonates admitted to the NICU of HUCSH, southern Ethiopia, 2021.

The proportion of neonatal death among admitted neonates was 15.4% (95% CI: 13%, 17%). The remaining were recovered [588 (75.15%)], refused [42 (4.97%)], referred [11 (1.3%)], and others [27 (3.2%)] as shown below (Figure 1).

### Model development and validation

Neonatal mortality was predicted using a prediction model that included sociodemographic, antepartum, intrapartum, and neonatal variables that were collected retrospectively. Bivariable binary logistic regression was used to get the association of potential determinants with neonatal mortality and to select candidate determinants for multivariable binary logistic analysis.

We incorporated the variables with *p* < 0.25 in the bivariable analysis into the multivariable model. A stepwise backward elimination technique with *p* < 0.1 was applied to fit the final reduced model.

Thirteen variables, namely, residence, gravida, ANC, birthplace, birth weight, mode of delivery, gestational age, 5th-minute Apgar score, amniotic fluid status (AF), respiratory distress (RD), PNA, sepsis, and breastfeeding, were predictor candidates for multivariable analysis.

In the multivariable analysis and simplified model, seven variables, namely, rural residence [*β* = 0.81,95% CI (0.37, 1.26)], primigravida [*β* = 0.64, 95% CI (0.16, 1.14)], low birth weight [*β* = 1.07, 95% CI (0.37, 1.57)], AF (stained) [*β* = 0.80, 95% CI (0.04, 1.42)], Apgar score (<7) [*β* = 1.21, 95% CI (0.63, 1.78)], PNA (yes) [*β* = 0.78, 95% CI (0.64, 1.34)], and breastfeeding (no) [*β* = 1.32, 95% CI (0.69, 1.94)], were predictors that remained significant factors and were included in the risk score development (Table 4).

**Table 4:** Coefficients and risk scores of each predictor included in the model to predict neonatal mortality of neonates admitted in the NICU of HUCSH, southern Ethiopia, 2021*.*

Predictor variables	Bivariable analysis		Multivariable analysis		Risk score
	*Β* (95% CI)	*p*-value	*β* (95% CI)	*p*-value	
Residence					
Rural	0.92 (0.52, 1.32)	<0.001	0.81 (0.37, 1.26)	<0.001***	1
History of medical					
Illness (yes)	0.20 (−1.08,0.68)	0.651	NA		
gravida (primi)	0.37 (0.05,0.801)	0.088	0.64 (0.16, 1.14)	0.001**	1
ANC (no)	0.99 (0.28, 1.70)	<0.01	0.81 (−0.14, 1.45)	0.093	
APH (yes)	0.24 (−1.03, 1.51	0.707	NA		
Birthplace (home)	1.04 (0.12, 1.97)	0.027	0.73 (−0.44, 1.82)	0.196	
Premature rupture of membrane (PROM) (yes)	−0.06 (−0.70, 0.578)	0.843	NA		
Complication during labor (yes)	0.02 (−0.52, 0.56)	0.932	NA		
BW (LBW)	1.02 (0.72, 1.32)	<0.001	1.07 (0.37, 1.57)	0.001**	2
Mode of delivery (CS)	0.35 (0.06, 0.760)	0.095	0.36 (−0.10, 0.84)	0.128	
Presentation (non-cephalic)	0.25 (−0.31, 0.81)	0.385	NA		
GA (<37 weeks)	0.99 (0.61, 1.37)	<0.001	0.29 (−0.30, 0.8)	0.337	
5th minute					
Apgar (≤7)	1.61 (1.17, 2.06)	<0.001	1.21 (0.63, 1.78)	<0.001***	2
AF status (stained)	0.41 (−0.15, 0.96)	0.15	0.80 (0.04, 1.42)	0.021*	1
RD (yes)	0.73 (0.24, 1.21)	<0.01	0.70 (0.09, 1.30)	0.056	
PNA (yes)	1.07 (0.62, 1.52)	<0.001	0.78 (0.04, 1.34)	0.019*	1
Sepsis (yes)	0.58 (0.03, 1.138)	0.01	0.28 (−0.27, 0.83)	0.318	
Breastfeeding (no)	1.55 (1.03, 2.07)	<0.001	1.32 (0.69, 1.94)	<0.001***	2

AF, amniotic fluid; ANC, antenatal care; Apgar, appearance, pulse rate, grimace, activity, respiration; APH, antepartum hemorrhage; PROM, premature rupture of membranes; BW, birth weight; GA, gestational age; NA, not applicable; PNA, perinatal asphyxia; RD, respiratory distress.

*P value <.05.

**P value <.01.

***P value <.001.

Model equation NM = 1/(1 + exp−(−5.4 + 0.8 * residence (rural) + 0.7 × amniotic fluid status (stained) + 0.8 * PNA + 1.3 × breastfeeding (no) + 0.6 × primigravida + 1 × low BW + 1.2 × 5th-minute Apgar score.

**Figure 2:**
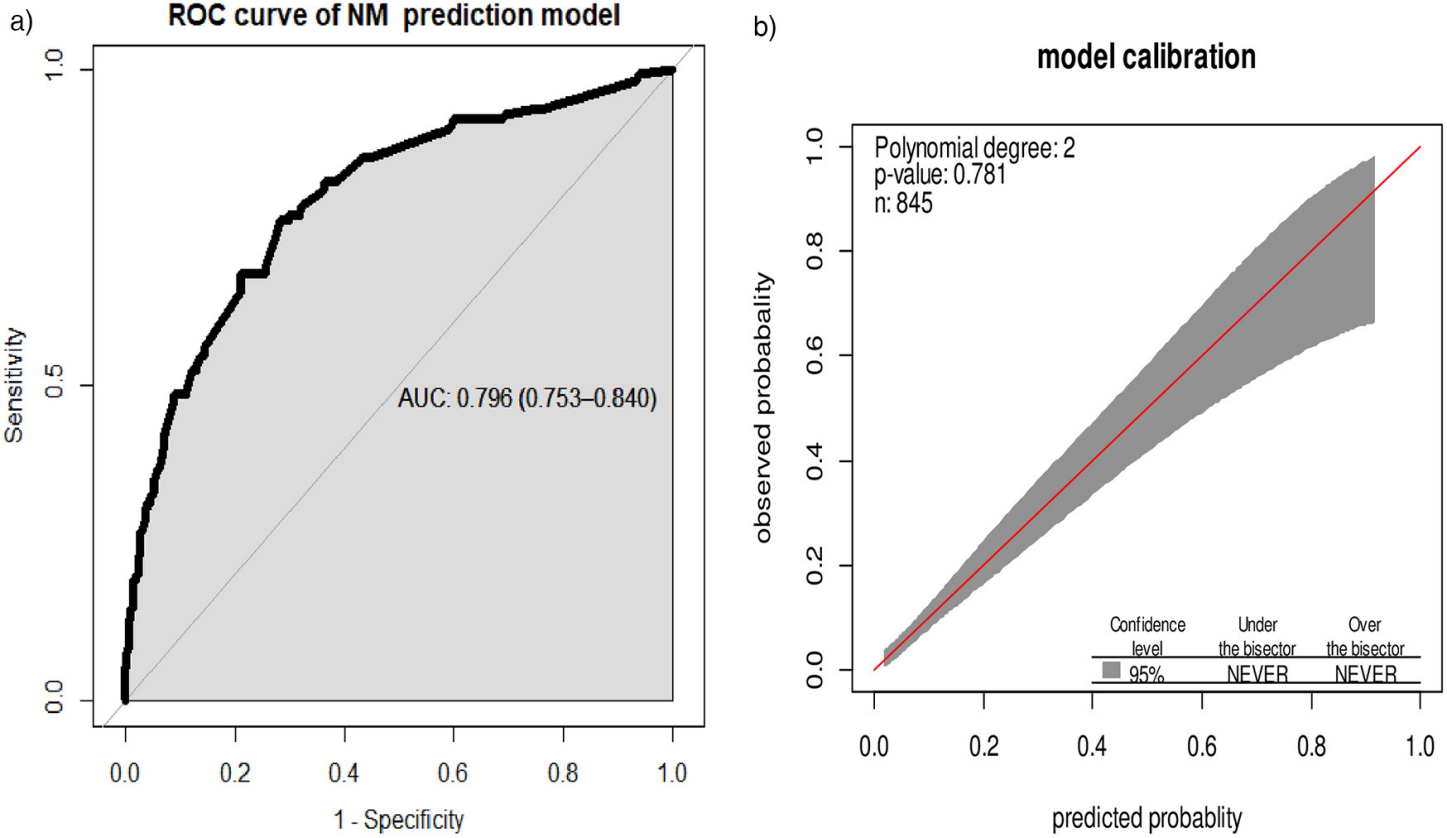
**(a)** Area under the ROC curve for the prediction model and **(b)** predicted vs. observed neonatal mortality probability in the sample. This analysis includes the calibration plot created using “givitiCalibrationBelt” in R programming.

The area under the ROC curve of the final model was 0.796 (95% CI: 0.753, 0.840). The calibration test showed a *p*-value of 0.781, and the Hosmer–Lemeshow statistic yielded *p* = 0.177 and *χ*² = 11.4, indicating that the model does not misrepresent the data (Figure 2).

**Figure 3:**
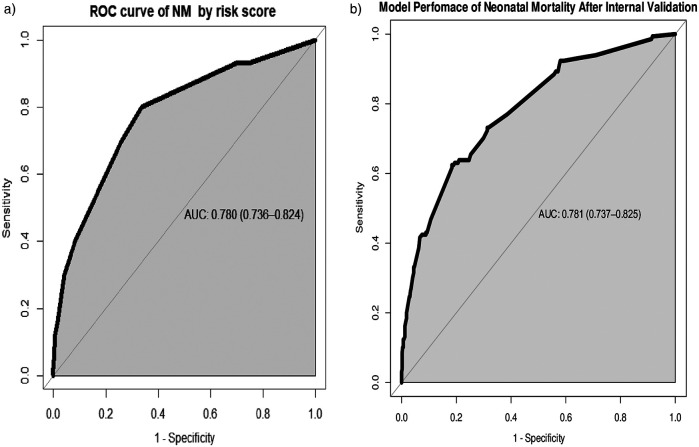
**(a)** Area under the ROC curve for the prediction model by risk score and **(b)** area under the ROC curve for the prediction model after bootstrapping.

While the AUC of the simplified risk score prediction model was 0.780 (95% CI: 0.736, 0.824). Internal validation of the model with the bootstrap technique showed no indication of undue influence by particular observations, with an optimism coefficient of 0.015, indicating minimal overfitting of the model to the data. Finally, the AUC result was 0.781 (95% CI: 0.737, 0.825) after internal validation (Figure 3).

The sensitivity across different cutoff point level thresholds showed that ≥3 had an optimal sensitivity of 80%, with a specificity of 66%, in predicting neonatal mortality in the NICU (Table 5).

**Table 5:** Sensitivity and specificity of the neonatal mortality at different cutoff points.

Cutoff points	High risk	Sensitivity	Specificity	LR+	LR−	PPV	NPV
≥2	499	0.93	0.30	1.3	0.2	0.19	0.96
≥3	240	0.80	0.66	2.3	0.3	0.30	0.94
≥4	182	0.70	0.74	2.7	0.4	0.33	0.93
≥5	59	0.40	0.92	4.8	0.6	0.47	0.89
≥6	29	0.30	0.95	7.3	0.7	0.57	0.88

We categorized neonates into high-risk (>3) and low-risk (<3) groups based on the risk score. Using the “Youden index,” the suggested cutoff to predict neonatal mortality yielded a sensitivity of 80%, a specificity of 66%, a positive predictive value of 30%, a negative predictive value of 94%, a positive likelihood ratio of 2.3, and a negative likelihood ratio of 0.3 (Table 5).

### Prediction density plot

**Figure 4:**
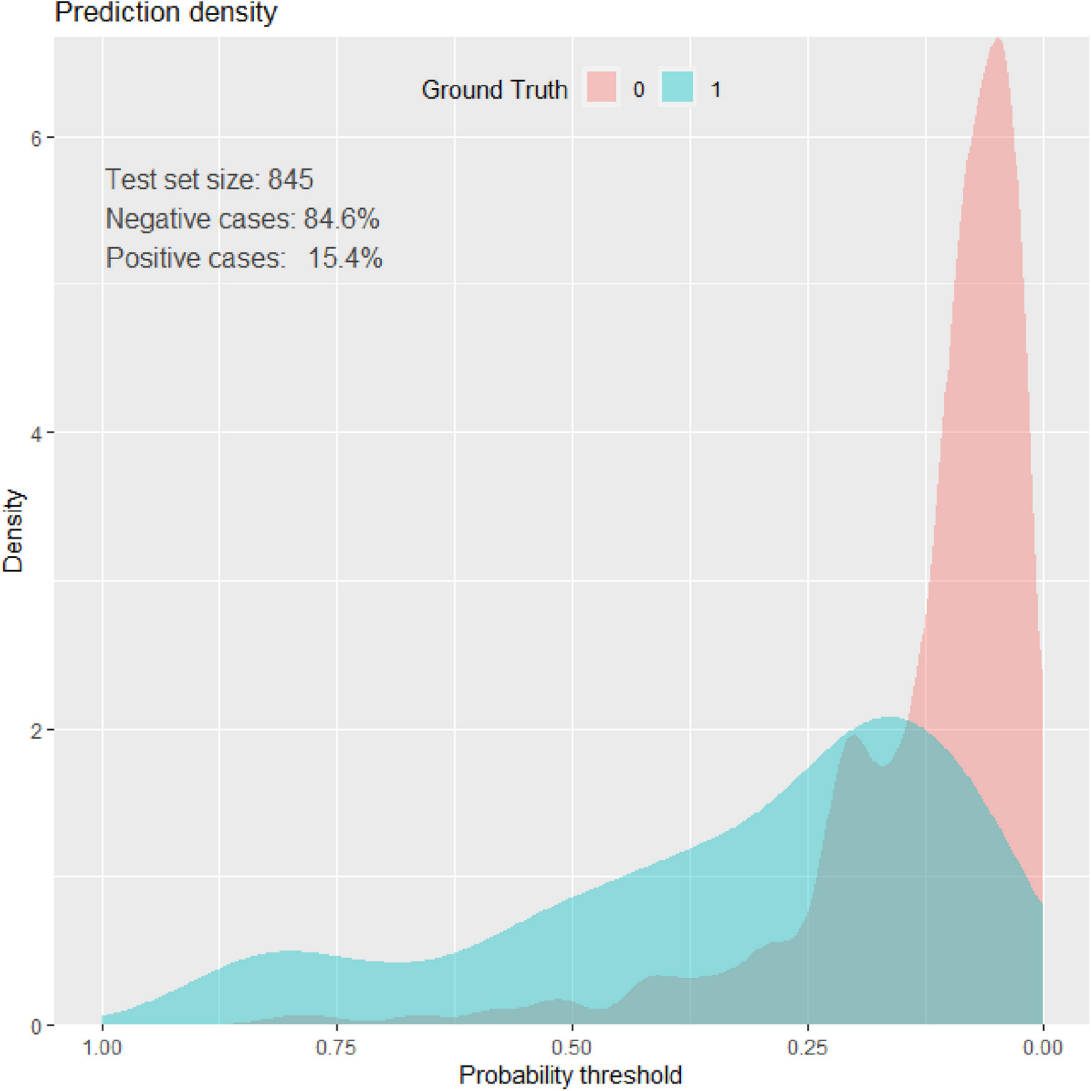
Prediction density plot of mortality among neonates admitted to the NICU of HUCSH.

Density plots demonstrate the distribution of key features among neonates who died (blue) and survived (red) during the NICU follow-up at HUCSH. The overlap of the curves demonstrated that no single variable can perfectly predict neonatal morality (Figure 4).

### Decision curve analysis

**Figure 5:**
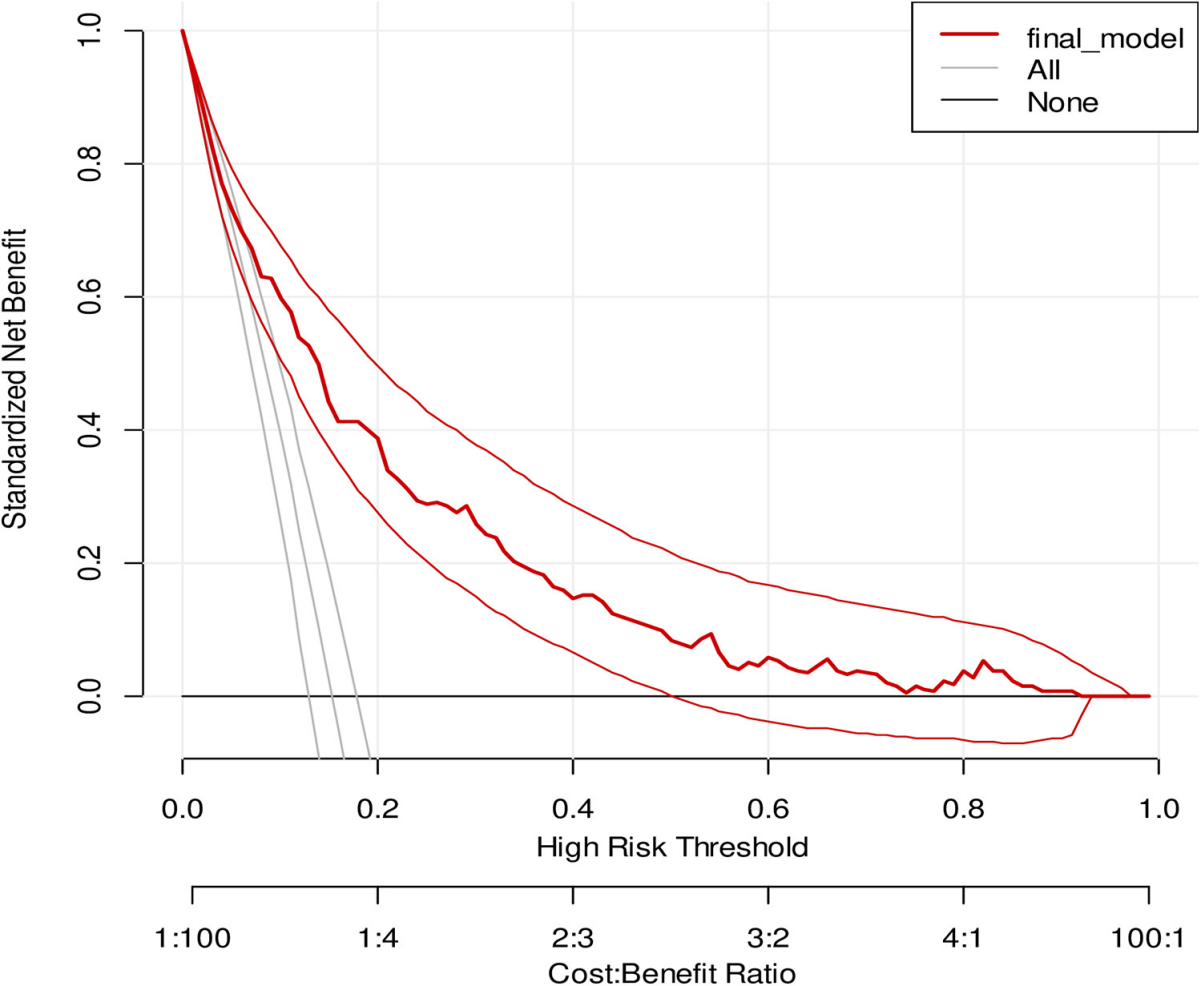
A decision curve plotting for the net benefit of the model against threshold probability and corresponding cost–benefit ratio.

The model has the highest net benefit across the whole range of threshold probabilities, showing that it has the most clinical and public health value, as seen in the graph below. As a result, utilizing the model to make decisions offers a higher net benefit than providing services routinely for everyone, regardless of their risk threshold (Figure 5).

### Risk classifications by risk score

We developed a simplified risk score from the model by rounding all regression coefficients in the reduced model resulting in a simplified prediction score as presented in (Table 4). The simplified score had comparable prediction accuracy with the original coefficients, with an AUC of 0.780 (0.736–0.824). The possible minimum and maximum scores developed from the model ranged from 0 to 10, respectively. The proportion of neonatal mortality by risk category was 7% for low-risk score category (<3), 28.7% for intermediate-risk category score (4–6), and 65.6% for high-risk group category (≥7) (Table 6).

**Table 6:** Risk classification of neonatal mortality using the simplified score (*n* = 845).

Risk score category	No. of participants	Incidence of mortality
Low (<3)	573 (67.8%)	40 (7%)
Intermediate (4–6)	240 (28.4%)	69 (28.7%)
High (≥7)	32 (3.8%)	21 (65.6%)
Total	845 (100%)	130 (15.4%)

Model equation for risk score = 1 × rural residence + 1 × amniotic fluid status (stained) + 1 × PNA + 2 × breastfeeding + 1 × primigravida + 2 × poor 5th-minute Apgar + 2 × low birth weight.

## Discussion

This study focused on the development and validation of a simplified risk score to predict neonatal mortality among neonates admitted to intensive care units in Southern Ethiopia using various predictors.

The current study shows the incidence of neonatal mortality at 15.4% among neonates admitted to the NICU. This result is higher than the studies conducted in the Wolaita Sodo referral hospital (37), at Nekemte Referral Hospital (38), Tigray region (11), Bangladesh (39), and rural surveillance centers of India, Bangladesh, and Nepal (40). On the other hand, it is lower than those in studies conducted at the University of Gondar (UOG) comprehensive specialized hospital, Amhara region referral hospitals, and Debre Markos referral hospitals (11, 13, 15).

This difference might be due to hospital-to-hospital service delivery, infrastructure differences, and some of the studies conducted in community surveillance centers. This result is almost consistent with the findings of studies conducted in Southern Ethiopia (41), Nigeria, and Nepal (42, 43). This may be due to the treatment and classification of disease based on WHO guideline recommendations and both countries are underdeveloping countries.

The study demonstrated optimal performance by integrating maternal, obstetrical, and neonatal characteristics to predict mortality among neonates admitted to the intensive care units in Southern Ethiopia. The prognostic factors included residence, perinatal asphyxia, breastfeeding status, primigravida, poor 5th-minute Apgar score, amniotic fluid status, and low birth weight.

In our study, PNA had a significant association with neonatal mortality. This finding is consistent with the result of a study conducted in the Wolaita Sodo referral hospital, the University of Gondar referral hospital, and seven hospitals of the Tigray region (11, 15, 37). This may be due to oxygen deficit at delivery can lead to severe hypoxic–ischemic organ damage in newborns followed by a fatal outcome or increases the probability of death.

In this study, failure to initiate breastfeeding was associated with increased neonatal mortality among admitted neonates. This is consistent with a study conducted in Southern Ethiopia (37). The finding is also supported by a systematic review of initiation of breastfeeding and neonatal mortality (44). The possible explanations might be immunological deficits, infection susceptibility, metabolic instability, and nutritional deficiencies. These physiological disruptions collectively predispose neonates to severe illness and death.

In this research, amniotic fluid status was also associated with neonatal mortality among admitted neonates. This finding is supported by the study conducted in eastern and northwestern Ethiopia (45, 46). The possible explanation might be that stained amniotic fluid, particularly when stained with meconium, is an indicator of potential fetal distress. This condition can lead to serious neonatal complications, including meconium aspiration syndrome (MAS), respiratory distress, sepsis, and other infections. These adverse outcomes can contribute to an increased risk of neonatal mortality.

Our study demonstrated that rural residence was a significant predictor of neonatal mortality, consistent with the findings of the EDHS 2019 and the National Newborn and Child Survival Strategy of Ethiopia (21, 24). This may be due to limited access to healthcare facilities, low utilization of maternal healthcare services, socioeconomic challenges, delays in receiving specialized care, disparities in healthcare resources, weak referral systems, and inadequate health infrastructure in rural settings.

Incorporating birth weight in forecasting neonatal mortality is the highest discrimination power. In this study, birth weight is one of the predictors to result in 78% of discrimination power. This is also supported by a study conducted in Gondar (47) and Bangladesh (39). Other studies have also shown the association between birth weight and neonatal mortality (14). This might be due to physiological vulnerabilities, including immature organ development, metabolic instability, and higher susceptibility to birth complications. Additionally, in resource-limited settings, the lack of specialized neonatal care worsens these risks.

Birth weight and 5th-minute Apgar score are also among the variables that affect the performance of our model. Some studies have also reported that these factors influence the predictive performance of the model (39, 47, 48). Fifth-minute Apgar score is one of the main predictors of neonatal mortality among neonates admitted to NICU, which has also been reported by a study conducted at the University of California (49).

This may be due to low Apgar scores in neonates reflecting physiologic immaturity rather than poor condition at birth and may also be caused by maternal drugs, infections, neurological diseases, and congenital anomalies.

The AUC of the combined predicting model is 0.796 (95% CI: 0.753, 0.840), and the final reduced and internally valid model yielded an AUC of 0.781 (95% CI: 0.737, 0.825), indicating that the model has a good predictive ability. This finding is comparable to a study conducted in Bangladesh where a simplified model (birth weight, GA, lethargy, cyanosis, non-cephalic presentation, and trouble suckling) predicted death with good discrimination [validation area under the receiver operating characteristic curve (AUC) 0.80 (95% CI: 0.73, 0.87)]. However, our model showed better performance than the additional simplified model from the same study (gestational age, non-cephalic presentation, lethargy, trouble suckling) that predicted death with moderate discrimination [validation AUC 0.74, 95% CI (0.66, 0.81)] (39).

Our model’s performance is lower than that reported in a study conducted in Mexico, which achieved an AUC of 0.92. This discrepancy may be due to the socioeconomic status of the countries (48). The performance is also lower in comparison with a prognostic study conducted in Nepal (43). This is mainly due to the study focusing on neonatal factors using Score for Neonatal Acute Physiology with Perinatal Extension (SNAPPE) scoring systems.

The performance score of our model is also higher than those reported in studies conducted in low- and middle-income countries, which analyzed data from population surveillance sites in India, Nepal, and Bangladesh. In these studies, the AUC was 0.59 (95% CI 0.58, 0.61) at the start of pregnancy and 0.73 (95% CI 0.70, 0.76) at the start of delivery. However, our model’s performance score was lower compared with the AUC measured at 5 min post-partum [AUC: 0.85, 95% CI (0.80, 0.89)] (40). This is maybe due to the inclusion of participants from different countries and substantial sociodemographic differences. In addition, the study incorporates a huge sample size.

After bootstrap internal validation, optimism corrected AUC was 0.781 (0.737, 0.825). Model optimism was estimated at 0.015 indicating minimal overfitting of the model to the data. This is lower than that reported in a study conducted at the University of Gondar (47). This discrepancy may be due to differences in predictors.

In our prediction score, using a cutoff point of 3 determined by the Youden index yielded an acceptable level of specificity (66%), sensitivity (80%), PPV (0.33), NPV (0.93), LR+ (2.3), and LR− (0.3) to predict neonatal mortality among neonates admitted to the NICU. The sensitivity observed in our study was higher compared with those reported in a study conducted in Bangladesh (39) and two studies in Mexico (48, 49) (80% vs. 65.3%, 66.7%, and 40%, respectively) but nearly similar to those reported in a study conducted in Gondar which is 81%. Specificity was higher in studies conducted in Bangladesh and Mexico (39, 49). On the other hand, it is lower than those reported in studies conducted in Gondar and Mexico (47, 48). The difference may be due to different cutoff values, different prognostic factors, the quality of intensive care, and differences in socioeconomic status.

This prognostic prediction model provides a significant potential for implementation in neonatal health services to predict mortality among neonates admitted to intensive care units. It enables healthcare providers to assess mortality risk early, prioritize high-risk neonates, and implement timely interventions such as enhanced monitoring and critical treatments. The model also supports standardized evaluations and evidence-based care, reducing variability in decision-making and improving neonatal outcomes.

In resource-limited settings, the model serves as a valuable tool for guiding the efficient allocation of medical resources, ensuring they are directed toward neonates at the highest risk. Additionally, it provides training opportunities for healthcare workers, enhancing their decision-making capabilities. On a broader scale, the model's predictive data can inform public health policies and optimize resource distribution.

A key strength of the study is that our prediction model was internally validated using a bootstrapping technique by simulating a huge sample to replace itself, resulting in a small optimism coefficient. We used an adequate number of outcomes, which enabled us to construct the prediction model using an adequate number of predictors. We developed the model using risk scores which enabled health professionals to calculate risk scores easily without advanced calculations. Lastly, our prediction model was constructed from easily available and measurable predictors that make it relevant in healthcare settings.

The limitation of the study was this study was conducted on admitted neonates who were at greater risk of death. Consequently, the NM reported from this study could be overestimated. In addition, it was conducted in a teaching referral hospital. We suggest external validation of this risk prediction model before adoption in diverse clinical and public health contexts to confirm its generalizability. Moreover, since it was a retrospective study, some important variables with strong predictive ability may not have been included.

## Conclusions

The overall incidence of neonatal mortality among neonates admitted to the NICU of HUCSH was high. We constructed a prediction model that has good performance for neonatal mortality, based on maternal characteristics and obstetrical and neonatal factors in the NICU of teaching hospitals in southern Ethiopia. The prediction score can help risk stratification during ANC, delivery, and postnatal period and in the identification of neonates at higher risk of mortality. High-risk groups can take precautions and early appropriate interventions. This is a feasible prediction score that can be readily applied by health professionals in the unit, contributing to the reduction of neonatal mortality and improvement of overall maternal and child healthcare.

## Data Availability

The original contributions presented in the study are included in the article/Supplementary Material, further inquiries can be directed to the corresponding author.
